# Impact of Depressive and Insomnia Symptoms on Memory Decline and Dementia Risk in Older Adults

**DOI:** 10.1093/geroni/igaf122.1964

**Published:** 2025-12-31

**Authors:** Sofia Liu, Claire Wang, Junxin Li

**Affiliations:** Johns Hopkins University, Baltimore, Maryland, United States; Johns Hopkins University, Baltimore, Maryland, United States; Johns Hopkins University, Baltimore, Maryland, United States

## Abstract

Depressive symptoms (DS) and insomnia symptoms (IS) often co-exist in older adults, yet their combined impact on cognition is understudied. This study investigates the association between baseline coexistence of depressive and insomnia symptoms on memory and dementia incidence over a one-year follow-up among community-dwelling older adults. We analyzed data from 4474 participants in the National Health and Aging Trends Study (NHATS) using Round (R) 12 (baseline) and R13 (follow-up). The sample included 70.48% aged 75 and older, 57.38% female, 64.30% White without dementia at baseline. Participants were classified as (1) neither condition (reference), (2) IS only, (3) DS only, and (4) both DS and IS at baseline. At follow-up, 41.72% experienced memory decline, and 2.12% converted to dementia. Generalized estimating equations models suggest those with both conditions showed a poorer memory at follow-up compared to the reference (B = -0.08, CI: -0.12, -0.04), adjusting for baseline sociodemographic and health factors. Logistic regression shows that individuals with only DS (OR = 3.80, CI: 1.71,8.45) and those with both conditions (OR = 2.39, CI: 1.31, 4.39) were associated with higher odds of developing dementia at follow-up compared to those with neither condition. The coexistence of depressive and insomnia symptoms is associated with worse cognitive performance over time and increases the risk of dementia. These findings highlight the potential compounding effect of coexisting depressive and insomnia symptoms on cognitive health. Addressing both conditions concurrently may mitigate dementia risks. Studies over longer periods of time are needed to confirm these findings and inform targeted interventions.

